# Unpacking the Implications of SARS-CoV-2 Breakthrough Infections on COVID-19 Vaccination Programs

**DOI:** 10.3390/vaccines10020252

**Published:** 2022-02-07

**Authors:** Tafadzwa Dzinamarira, Nigel Tungwarara, Itai Chitungo, Munashe Chimene, Patrick Gad Iradukunda, Moreblessing Mashora, Grant Murewanhema, Gallican Nshogoza Rwibasira, Godfrey Musuka

**Affiliations:** 1 School of Health Systems & Public Health, University of Pretoria, Pretoria 0002, South Africa; 2 ICAP, Columbia University, Harare P.O. Box MP167, Zimbabwe; gm2660@cumc.columbia.edu; 3 Department of Health Studies, University of South Africa, Pretoria 0002, South Africa; 34846751@mylife.unisa.ac.za; 4 Chemical Pathology Unit, Department of Medical Laboratory Sciences, Faculty of Medicine and Health Sciences, University of Zimbabwe, Harare P.O. Box MP167, Zimbabwe; ichitungo@medsch.uz.ac.zw; 5 Department of Health Sciences, Africa University, Mutare P.O. Box 1320, Zimbabwe; munashe24@gmail.com; 6 London School of Hygiene and Tropical Medicine, University of London, London WC1E 7HU, UK; gadpatrickiradukunda@gmail.com; 7 Department of Public Health, Mount Kenya University, Kigali P.O. Box 5826, Rwanda; mcmashora@gmail.com; 8 Unit of Obstetrics and Gynaecology, Department of Primary Health Care Sciences, Faculty of Medicine and Health Sciences, University of Zimbabwe, Harare P.O. Box MP167, Zimbabwe; gmurewanhema@gmail.com; 9 Rwanda Biomedical Centre, Ministry of Health, Kigali P.O. Box 7162, Rwanda; gallican.rwibasira@rbc.gov.rw

**Keywords:** SARS-CoV-2, vaccination, breakthrough infections

## Abstract

Despite an array of preventive global public health interventions, SARS-CoV-2 has continued to spread significantly, infecting millions of people across the globe weekly. Newer variants of interest and concern have continued to emerge, placing the need for policymakers to rethink prevention strategies to end the pandemic. The approval of SARS-CoV-2 vaccines for public health use in December 2020 was seen as a significant development towards pandemic control and possibly ending the pandemic. However, breakthrough infections have continued to be observed among the ‘fully vaccinated’, and the duration and sustainability of vaccine-induced immunity has remained a topical public health discourse. In the absence of accurate public health communication, the breakthrough infections and waning immunity concepts have potential to further compound vaccine hesitancy. With this viewpoint, we discuss breakthrough SARS-CoV-2 infections, waning immunity, the need for COVID-19 booster shots, vaccine inequities, and the need to address vaccine hesitancy adequately to propel global vaccination programs forward.

## 1. Introduction

Severe Acute Respiratory Syndrome Coronavirus 2, the causative agent for COVID-19, continues to pose a significant global health threat. Since it was discovered in China in December 2019, became an international public health emergency in early 2020, and was declared a pandemic by the World Health Organization (WHO) in March 2020, the virus has continued spreading inexorably [1]. Despite an array of preventive global public health interventions, the virus has continued to spread significantly, infecting millions of people across the globe weekly. WHO weekly epidemiological reports show that as of 9 January 2022, the cumulative global number of confirmed COVID-19 cases had risen to 304,350,207 while the cumulative fatalities from COVID-19 stood at 5,482,865. Moreover, 15,154,666 new cases were reported globally seven days before 9 January 2022 [1].

Newer variants of concern (VOC) have continued to emerge, placing the need for public health stakeholders to rethink prevention strategies to end the pandemic. The WHO has defined a VOC as exhibiting significantly increased transmissibility that can change the known epidemiological disease pattern or has the potential to evade working preventive public health measures such as vaccination [2]. The approval of SARS-CoV-2 vaccines for public health use in December 2020 was seen as a significant development towards pandemic control and possibly ending the pandemic [3]. However, breakthrough infections have continued to be observed among the fully vaccinated, and the duration and sustainability of vaccine-induced immunity have remained a topical public health discourse. Anti-SARS-CoV-2 antibodies have been noted to play a significant role in breakthrough infections, with recently acquired antibodies from recent vaccination being less protective than refined antibodies generated from memory B-lymphocytes after waning of the initial antibody response. In the absence of well-coined public health communication, the breakthrough infections and waning immunity concepts can drive or worsen vaccine hesitancy. With this viewpoint, we discuss breakthrough SARS-CoV-2 infections, waning immunity, the need for COVID-19 booster shots, vaccine inequities, and the need to address vaccine hesitancy adequately to propel global vaccination programs forward.

## 2. SARS-CoV-2 Related Breakthrough Infections

SARS-CoV-2 vaccines are believed to be one of the most successful strategies in the fight against the ongoing COVID-19 pandemic. This is because studies have demonstrated their effectiveness in reducing new infections, moderate and severe disease, hospitalization, and death among infected individuals [4,5]. However, there have been reports of infection among the fully vaccinated, termed breakthrough infections. These are SARS-CoV-2 infections in an individual who has completed all the required doses of a COVID-19 vaccine with a typical 14-day lag period [6,7]. Most breakthrough infections were asymptomatic or mild in Israel, although persistent symptoms did occur [6]. There is minimal information on the risk factors associated with breakthrough infections after complete vaccination [8]. However, among others, being of elderly age, the presence of comorbidities such as type 2 diabetes mellitus and cardiovascular disease, being on immunosuppressive therapies, and living with HIV/AIDS have been noted as significant risk factors for breakthrough infections. Among those with cancers, those with hematological malignancies are at higher risk compared to non-hematological malignancies. These infections have been linked to waning immunity and strain-specific decline in vaccine effectiveness, termed immune escape as was noticed with the surge of infections with the Delta variant [7,9]. The Delta variant has been reported to contain mutations in the spike protein that are divergent from the three prior variants of concern, Alpha (B.1.1.7), Beta (B.1.351), and Gamma (P.1). These spike mutations that increase infectivity could enable the virus to rapidly attach and infect respiratory epithelial cells, avoiding the relatively sparse neutralizing antibodies in the mucosa, thus allowing for virus breakthrough [10].

Some scholars have argued on what qualifies as a COVID-19 breakthrough infection. For example, Schieffelin and colleagues suggested that the term breakthrough infection implies that the virus broke through a protective barrier provided by the vaccine [11]. They base this on the fact that vaccines do not prevent nasal (upper airway) infection, which is the site used to confirm infections in routine monitoring. Instead, the current vaccines elicit anti-spike IgG and T cell responses that can be detected in peripheral blood. There is paucity of data on whether these intramuscular vaccines also elicit respiratory tract-specific immune reactions such as the generation of tissue-resident memory B cells and T cells. They suggest a definition for breakthrough infection as a positive SARS-CoV-2 PCR test from the respiratory tract and documentation of lower respiratory tract disease in fully vaccinated people with mild to asymptomatic cases not classified as breakthroughs [11]. Studies have reported a higher risk of COVID-19 breakthrough infections with possibilities of severe symptoms in immunocompromised individuals with conditions like HIV, rheumatoid arthritis, and solid organ transplant [7,12], as well as those on biological therapies.

## 3. COVID-19 Vaccine-Induced Immunity and Waning Immunity

With the COVID-19 pandemic approaching the end of its second year, immunologists and vaccinologists have a tough challenge in characterizing the durability of the protective immunity conferred by the COVID-19 vaccines [13,14]. The decline in the vaccine-induced antibodies over time, referred to as waning immunity, varies with diseases and the vaccine type [15]. The durability of vaccine effectiveness, especially against the more transmissible SARS-CoV-2 variants, has been questioned, especially in some in high-income countries that have seen a resurgence of COVID-19 despite their advanced vaccination programs [16]. The concept of waning immunity in vaccinated individuals is not a new phenomenon. For example, as discussed by Hamami and colleagues, individuals with mumps vaccination can become susceptible to infection as time from vaccination extends [17]. SARS-CoV-2 waning immunity has been cited as a possible reason for reduced vaccine effectiveness against the Delta (B.1.617.2) variant responsible for the resurgence of the COVID-19 outbreak in Israel six months following a mass vaccination BNT162b2 vaccine in December 2020 [18,19].

Despite this possibility of waning immunity, studies have shown that vaccines can still provide high levels of protection against symptomatic disease and severe disease and death caused by the Delta variant breakthrough infections. In a retrospective cohort study conducted in the United States of America (USA), the BNT162b2 vaccine effectiveness against SARS-CoV-2 infections waned during the first six months, and effectiveness against hospital admissions in all age groups did not wane throughout the study [20]. This observational study estimated vaccine effectiveness by comparing rates of SARS-CoV-2 infection and COVID-19- related hospital admissions among fully vaccinated and partially vaccinated individuals to those who were unvaccinated [20].

There are challenges in using the rise of COVID-19 infections in communities that have been vaccinated as an indicator for vaccine effectiveness. Some dynamics vary from one country to another, such as prevalence, behavior, and circulating variants, which makes comparing immune protection changes over time less reliable [16]. For instance, the study in Israel was prone to biases that could have arisen from the non-randomization of the timing of vaccination, risk factors of exposure to COVID-19, and the tendency to seek testing as a confounder to the association between time since vaccination and infection [16]. To assess vaccine effectiveness against infection, there is a need for systematic random sampling and consideration of a comprehensive range of measured and unmeasured confounders. A study close to this in Croatia tested the samples from BioNTech/Pfizer fully vaccinated nursing home residents with no history of COVID-19 infections for the presence of neutralizing antibodies using a vaccine neutralizing test. The findings demonstrated that almost half (46%) of the participants had a negative or low positive titer six months after being fully vaccinated, suggesting the humoral immunity waning [21]. The studies cited in our present discussion show waning immunity as a function of time from complete vaccination translating to time to reinfection. This is fundamental to numerous aspects of public health decision making that encompass the provision of booster doses and continuous adherence to non-pharmaceutical preventive measures. This evidence around the probability of waning of protection against SARS-CoV-2 infections, especially with the emerging variants, has led to countries considering using booster doses to enhance population protection and reduce transmission.

## 4. The Need for Booster Jabs

The need for booster doses for COVID-19 has been debated, with some global health authorities coming out publicly against the idea [22]. The approach to have booster doses has been based on the argument that the effectiveness of the vaccines against SARS-CoV-2 infections wanes over time as has been seen with other vaccines [23]. Vaccine boosters have been in use worldwide for many years, with some administered annually (intra-seasonal) for influenza, commonly known as flu jabs, as a measure to address the waning of previously administered doses. There has been evidence in the USA to suggest that the effectiveness of the inactivated influenza virus wanes during a single season [24]. Influenza viruses, similar to the SARS-CoV-2, undergo changes referred to as antigenic shift and antigenic drift, which raise the need for periodically updating the vaccines to retain effectiveness.

Evidence from some research, with findings still under peer review, suggests that the Omicron variant is associated with reduced neutralizing antibody responses following two doses of vaccine, which is reversed by a booster dose or hybrid immunity from a combination infection and vaccination [25]. However, another school of thought suggests that the messenger RNA (mRNA) vaccines’ reduced effectiveness is attributable to the short time spacing between the first and second doses, which resultantly reduced their lasting protection, thereby creating a need for a third dose [23]. This is further explained by the fact that immune response rises higher and faster after a third mRNA vaccine administered 8 months after the second dose than it would after the first two doses administered only a few weeks apart [23]. The short time spacing in the first two vaccine doses in the United Kingdom was mainly driven by the need to ease lockdown restrictions and to have as many adults fully vaccinated as the Delta variant emerged [26,27].

The campaign to have booster doses does not only stem from the need to reduce infections but also to reduce severe illnesses, which will put a lot of pressure on the public health systems around the world, which are still to recover from the effects of the pandemic fully. In one study in Israel which involved participants who were 60 years of age or older who had received two doses of the BNT162b2 vaccine at least five months earlier, the rates of confirmed COVID-19 infections and severe illness were significantly lower among those who had received a booster dose of the BNT162b2 vaccine [28]. Owing to the projected rise in COVID-19 infections during the winter season, some developed countries such as the USA, UK, and other European Union member states started to roll out booster vaccination programs. The UK is speeding up their booster rollout and more than 30 million people have received the booster dose as of 21 December 2021 to keep the immunity levels high with the emergence of the highly transmissible Omicron variant [29,30].

The need for vaccine booster doses has been highlighted by a recent study conducted among healthcare workers. It was noted that individuals who had been infected with COVID-19 infection for more than three months before acquiring a second dose had higher antibodies following six months of follow-up [31]. More importantly, Moderna vaccines have been demonstrated to significantly boost the immunity among individuals who received two shots of AstraZeneca and Pfizer vaccines, with antibodies being increased by 32 and 11.5 times, respectively [32]. Hence, considering the high transmissibility of the new variants, booster vaccine programs are believed to help reduce hospitalizations and deaths and prevent health systems from being overburdened. Nonetheless, some commentators have argued the ethics of high-income countries giving additional vaccine doses to their populations when much of the world’s population, especially in low-to-middle income countries of sub-Saharan Africa, is yet to receive its first dose [9,16,25,29].

## 5. COVID-19 Vaccine Inequities

At the inception of global COVID-19 vaccination programs, it was anticipated that richer countries and those that were part of the COVAX initiative would advance with their vaccination programs ahead of poorer countries, especially those from sub-Saharan Africa, several of which were outside this agreement. Three months after vaccine approvals and rollout, over one billion shots of SARS-CoV-2 vaccines had been administered, but by far the most significant majority in developed countries [33]. The World Health Organization (WHO) has been leading the campaign against COVID-19 vaccine hoarding and nationalization by more prosperous nations, leaving the 30 poorest countries in the world having only fully vaccinated approximately 2% of its population [22]. These extreme disparities in access to vaccines between the high-income and low-income countries are believed to have created the ideal conditions for the ongoing evolution of the SARS-CoV-2, evidenced by highly transmissible variants such as Delta and Omicron [16,25]. The emergence of the Omicron variant is further exacerbating the unmet need for vaccines in developing countries as the high-income countries are now diverting attention and limited vaccine supplies from the urgent need for primary vaccination to use in booster vaccinations.

An analysis conducted by the People’s Vaccine Alliance showed that between 11 November and 21 December 2021, the EU, UK, and US have received 513 million doses of vaccines while countries in Africa received just 500 million throughout the whole of 2021. The UK set a target to administer 1 million COVID-19 vaccine doses per day in response to the Omicron variant which translates to 1.46 percent of the UK population. If every country could deliver the same vaccination rate, it would only take 68 days to have every person who needs a first dose vaccinated, translating to the attainment of global coverage by February 2022 [34]. With the evidence that breakthrough infections are more common in the elderly, those with comorbidities and the immunocompromised [7,9,12], it is critical to promote targeted interventions to enhance the protection of the vulnerable populations while availing resources to the yet to be fully vaccinated population. Despite the prediction by experts that everyone fully vaccinated will require a booster dose, it would have been reasonable to give these to targeted populations in whom the evidence shows they are probably needed. This approach would make vaccines available to countries where they are most needed [35]. The countries that have limited vaccine coverage are now acting as the incubators for the virus as the virus has more hosts to infect and more time to mutate. Considering the limited evidence on the effectiveness of the booster vaccines against the new variants [36] and the overwhelming evidence of the effectiveness of primary vaccination [16], more resources should be channeled towards ensuring universal global vaccine coverage. As the WHO emphasizes, no one is safe until all are safe and with COVID-19 being a pandemic (global epidemic), a global strategy will be key to successful outcomes.

As with any other infectious disease, control of the spread of the SARS-CoV-2 virus relies upon several personal protective and social measures, including handwashing, mask-wearing, and physical distancing [37,38]. Some studies have reported post-vaccination behavioral changes in prevention measures used before vaccination. A cross-sectional study in Southern Ethiopia among health workers found a significant reduction in adherence to protective mechanisms after taking the first round of the COVID-19 vaccine [39]. The protracted battle against the COVID-19 has precipitated pandemic fatigue across several populations, resulting in widespread human complacency. Adherence to non-pharmaceutical preventative measures and equitable vaccinations is a cost-effective and sustainable way to curb new variants, which will reduce the need to respond with booster vaccines. Until herd immunity to COVID-19 is reached, public health preventive measures will remain the first choice measures in infection prevention and control [37] especially considering that in some countries in resource-limited settings, the populations are still largely unvaccinated. Moreover, even in resource-rich countries, vaccine hesitancy remains a significant public health challenge in some population segments.

## 6. Vaccine Hesitancy

Vaccine hesitancy refers to individual-level reluctance to receive vaccines [40]. It has been defined as refusal or delay in the uptake of vaccines despite their availability. The World Health Organization posit the 3Cs—convenience, complacency, and confidence—for not getting vaccinated. Convenience is a function of availability and access for those already willing to get vaccinated, while complacency and confidence relate to individuals’ perceptions and attitudes towards vaccines. Agency (vaccine and disease) factors inform the perception of vaccine safety and effectiveness and perceived susceptibility to the disease [41]. People would not get vaccinated because they think they do not need it (complacency) or are concerned with vaccines’ effectiveness and safety (confidence). Confidence is a function of how individuals acquire or receive information on vaccines and their efficacy.

The main drivers of vaccine hesitancy are misinformation and disinformation regarding the efficacy, vaccine production and composition, and adverse effects of vaccination; all these factors conspire to limit patient understanding and overall acceptance [40]. Most anti-vaccination campaigners argue against vaccination efficacy and utility and further query the unprecedented rapid development of COVID-19 vaccines [42], factors which collude to undermine public confidence. This emerging understanding of COVID-19 immunology on waning vaccine effectiveness and breakthrough infections are pivoted by anti-vaxxer and conspiracy theorists as further proof of vaccine ineffectiveness denting public confidence in vaccination. This highlights that information on vaccination matters contributes to vaccine hesitancy and threatens to undermine the success of COVID-19 vaccination programs. A collaborative approach by governments, health policy makers and media (mainstream and social) is required to provide precise and transparent information to dispel myths and misconception about vaccines [43].

## 7. The Need for Enhanced Public Health Messages Related to COVID-19 Vaccines

COVID-19 remains a global public health problem that calls for a joint approach to controlling the spread and emergence of transmissible variants. The rise in infections for the first time during the vaccine era is proof that the fight against COVID-19 cannot be won by vaccines alone but requires multi-pronged approaches that combine vaccination and public health preventive measures. Public health preventive measures such as hand hygiene, mask-wearing, and maintenance of physical distance will remain the cornerstone in reducing the spread of infection and should continue to be emphasized in all health promotion messaging around the world. Although vaccines will not protect people completely from COVID-19 diseases, they have been proven to significantly reduce the risk of severe symptoms, hospitalization, and death in those fully vaccinated. To fight against vaccine hesitancy, the conspiracy theories highlighted on COVID-19 vaccine need to be disvalued by comprehensive and clear communication of the action mechanism for the current approved and emergency use COVID-19 vaccines concerning outcomes settled during the conducted clinical trials. Widely availing the safety and effectiveness of the currently available and future vaccines to scientific communities and the public remains indispensable for boosting vaccine confidence and uptake. Unfortunately, the use of some vaccines such as the Sinovac and Sinopharm commenced when such data were not accessible, which could have contributed to the vaccine specific hesitancy around them. An online survey carried out in Canada suggested that providing information on the effectiveness of less-preferred vaccines at preventing death from COVID-19 is associated with more confidence in their efficacy and less vaccine-specific hesitancy [44]. Despite the abundant scientific evidence on the effectiveness of vaccines, there is evidence to suggest that humans are not good at understanding the statistical form the evidence is present in [45]. There is need to develop public health messaging that employs narrative techniques that highlight disease severity and the benefits of vaccinations.

## 8. Call to Action: Capacitate Resource-Limited Settings for Vaccine Production, Smooth Vaccine Distribution, and Genomic Sequencing

Allowing the manufacture of generic vaccines in resource-limited settings, especially those of sub-Saharan Africa will substantially reduce the costs associated with importing vaccines on a continent with scarce foreign currency reserves and reduce the logistics related to long-distance transportation. However, this is not an easy task as it requires substantial equipment and human resources investments on the African continent. Closer cooperation between the high-income and low-income countries is required and granting permission to manufacture generics by the big pharma. This will significantly depend on all players involved recognizing and reiterating the remarks by the WHO that no one is safe until all are safe.

Similarly, it is essential to capacitate the low-resource settings of sub-Saharan to perform frequent genetic sequencing [46], including among individuals with breakthrough infections to timely detect emerging variants, some of which may be variants of concern. Regular genomic sequencing must be perceived as an essential element of SARS-CoV-2 surveillance globally to detect these variants and inform the effectiveness of ongoing vaccination programs and the need to modify them periodically. Similar to vaccine manufacturing, this requires capacitation in sub-Saharan Africa through collaboration with international partners, including research institutions in developed countries [47].

Additionally, there is a need to ensure in-country equitable distribution of vaccines to ensure wider reach, including marginalized areas. This is especially pertinent in sub-Saharan Africa, where substantial proportions of the population are rural, with poor road networks and experience challenges in reaching health facilities, even for routine healthcare services. As the campaigns to accelerate vaccination programs and propel countries towards achieving their herd immunity thresholds gather momentum, strategies must be included, including mobile vaccination clinics in place. Alongside this, there is a great need to address vaccine hesitancy on the African continent consider vaccination for special groups such as adolescents, pregnant and breastfeeding people, and other groups that might have been left out.

## 9. Conclusions

Boosting global vaccine confidence and resultant uptake increase will significantly reduce strains on public health systems, especially in the resource-limited settings owing to less severe disease and reduced hospitalizations among the vaccinated. An increase in vaccine uptake will also result in reduced breakthrough infections and escape mutations, bringing an appreciable level of control to the pandemic. This could boost the evident low hospitalization and mortality rates despite the Omicron wave and mitigate breakthrough infections related to vaccine hesitance. However, as many high-income countries have rolled out rapid booster vaccination campaigns to deal with the waning immunity, the world will remain at risk of further pandemics if there are many countries with unvaccinated populations.

## Data Availability

Not applicable.
